# Hypernatremia in neurointensive care: results of a 5-year prospective study

**DOI:** 10.1186/cc9764

**Published:** 2011-03-11

**Authors:** V Spatenkova, A Kazda, P Suchomel

**Affiliations:** 1 Regional Hospital, Liberec, Czech Republic; 2 1st Faculty of Medicine, Charles University, Prague, Czech Republic

## Introduction

Hypernatremia is a common medical complication in neurointensive care that is associated with worse outcome. It can be caused by water diuresis due to anti-diuretic hormone insufficiency in central diabetes insipidus (cDI) or from different mechanisms: osmotherapy, furosemide or renal failure. The aim of this prospective study was to analyse hypernatremias in neurointensive care over a period of 5 years.

## Methods

We evaluated all hypernatremias defined as serum sodium (SNa^+^) >150 mmol/l in patients with acute brain disease hospitalised in the neurologic-neurosurgical care unit (NNICU). cDI was diagnosed according to serum and urine osmolality, hourly diuresis, electrolyte-free water clearance (EWC) and response to desmopressin. The remaining hypernatremias were called non-cDI. We compared these groups in Glasgow Coma Scale (GCS) on onset of hyponatremia, incidence of cerebral complications, Glasgow Outcome Scale (GOS) upon discharge from the NNICU and mortality in the NNICU, and EWC.

## Results

There were 133 hypernatremic patients (mean SNa^+ ^154.9 ± 4.5 mmol/l) with mean age 60.6 years; male 72; diagnoses: stroke 88 patients, tumour 19 patients, trauma 19 patients, infection four patients, others three patients. The mean GCS on onset of hypernatraemia was 9.4 ± 4.3, the mean GOS upon discharge from the NNICU was 2.4 ± 1.2. We diagnosed cDI in 16 patients, the majority (117 patients) was filed as the non-cDI group. Patients with cDI had significantly higher SNa^+ ^(160.1 ± 8.4 mmol/l, *P *< 0.001), diuresis (*P *< 0.001), EWC (*P *< 0.001), mortality in the NNICU (*P *= 0.012) than patients in the non-cDI group. There were no differences in GCS (*P *= 0.192), GOS (*P *= 0.079), cerebral complications (*P *= 0.809), and anti-edematic therapy (*P *= 0.221). Patients in the non-cDI group (SNa^+ ^154.4 ± 3.4 mmol/l) received more diuretics (*P *= 0.001) and 18 patients had renal failure.

## Conclusions

In this study cDI was not a common type of hypernatremia in neurointensive care, but it had higher mortality in the NNICU than other types of hypernatremias, which are caused mostly by diuretics and by renal failure.
